# Up-regulation of Toll-like receptors 2, 3 and 4 in allergic rhinitis

**DOI:** 10.1186/1465-9921-6-100

**Published:** 2005-09-07

**Authors:** Mattias Fransson, Mikael Adner, Jonas Erjefält, Lennart Jansson, Rolf Uddman, Lars-Olaf Cardell

**Affiliations:** 1 Laboratory of Clinical and Experimental Allergy Research, Department of Oto-Rhino-Laryngology, Malmö University Hospital, Lund University, Malmö, Sweden; 2 Department of Experimental Medical Science, Lund University Hospital, Lund University, Sweden; 3 AstraZeneca R&D, Lund, Sweden

## Abstract

**Background:**

Toll-like receptors enable the host to recognize a large number of pathogen-associated molecular patterns such as bacterial lipopolysaccharide, viral RNA, CpG-containing DNA and flagellin. Toll-like receptors have also been shown to play a pivotal role in both innate and adaptive immune responses. The role of Toll-like receptors as a primary part of our microbe defense system has been shown in several studies, but their possible function as mediators in allergy and asthma remains to be established. The present study was designed to examine the expression of Toll-like receptors 2, 3 and 4 in the nasal mucosa of patients with intermittent allergic rhinitis, focusing on changes induced by exposure to pollen.

**Methods:**

27 healthy controls and 42 patients with seasonal allergic rhinitis volunteered for the study. Nasal biopsies were obtained before and during pollen season as well as before and after allergen challenge. The seasonal material was used for mRNA quantification of Toll-like receptors 2, 3 and 4 with real-time polymerase chain reaction, whereas specimens achieved in conjunction with allergen challenge were used for immunohistochemical localization and quantification of corresponding proteins.

**Results:**

mRNA and protein representing Toll-like receptors 2, 3 and 4 could be demonstrated in all specimens. An increase in protein expression for all three receptors could be seen following allergen challenge, whereas a significant increase of mRNA only could be obtained for Toll-like receptor 3 during pollen season.

**Conclusion:**

The up-regulation of Toll-like receptors 2, 3 and 4 in the nasal mucosa of patients with symptomatic allergic rhinitis supports the idea of a role for Toll-like receptors in allergic airway inflammation.

## Background

Toll-like receptors (TLRs) have recently emerged as key receptors of the innate immune system. They recognize specific pathogen-associated molecular patterns initiating a host defense response [1]. The upper airway encounters potential pathogens like bacteria and viruses in inspired air, and the discovery of TLRs on epithelial cells suggests that the epithelium has a role in the mucosal immune system [2-4]. Activation of TLR-dependent signaling pathways results in the expression of effector molecules, such as cytokines and chemokines, which contributes to the activation of the antigen-specific adaptive immune response [5]. The discovery that microbial components and endogenous ligands are recognized by TLRs may explain how generated signals can affect the initiation, maintenance and progression of inflammatory diseases [6]. Several authors have recently suggested a role for TLRs in the pathophysiology of allergic rhinitis and asthma, thus indicating the importance to study their expression during allergic airway inflammation [7,8].

Ten distinct TLRs have been described in humans, expressed in various combinations in cells of the immune system as well as in other cell types [9,10]. mRNA of all ten TLRs has been described in human nasal airway tissue, but protein verification using Western blot analysis or immunohistochemistry is still lacking for most TLRs in the nose [2,11]. In cultured airway epithelial cells, mRNA and corresponding proteins have been demonstrated for TLR2, TLR3, TLR4 and TLR5 [4,12-14]. TLR2 has also been demonstrated in the lower respiratory tract using immunohistochemistry and both TLR2 and TLR4 have been shown in alveolar epithelial cells with real-time PCR and flow cytometry [15,16]. No difference in the sinonasal mRNA expression of TLR2 and TLR4 could be demonstrated when patients with chronic sinusitis and/or nasal polyps were compared with healthy controls [3]. In another study, TLR4 immunoreactivity was detected in nasal mucosa from both children and adults irrespective of atopy status, revealing a higher expression level among children [17].

The present study was designed to quantify mRNA and protein expressions of TLR2, TLR3 and TLR4 in the nasal mucosa of patients with seasonal allergic rhinitis, before and after pollen exposure, and to compare these findings with data derived from healthy volunteers.

## Methods

### Skin prick test

Skin prick tests (SPTs) were performed with a standard panel of 10 common airborne allergens (ALK, Copenhagen, Denmark) including pollen (birch, timothy and artemisia), house dust mites (*D. Pteronyssimus and D. Farinae*), molds (*Cladosporium and Alternaria*) and animal allergens (cat, dog and horse). SPTs were performed on the volar side of the forearm with saline buffer as negative and histamine chloride (10 mg/ml) as positive control. The wheal reactions were measured after 20 min and designated as 4+, 3+, 2+, 1+ or 0 depending on the size in relation to histamine and saline [18].

### Subjects

The study included 42 patients (22 women) with symptomatic birch and/or grass pollen induced intermittent allergic rhinitis and 27 healthy volunteers (13 women), serving as controls. The median (range) age of patients and controls was 36 (18–68) and 27 (16–50) years, respectively. All control patients were healthy and the same goes for the rhinitis patients, with the exception of their allergy. None of the participants were subjected to any other type of surgery than the nasal biopsy described in the research protocol.

The diagnosis of birch and grass pollen induced allergic rhinitis was based on a positive history of intermittent allergic rhinitis for at least 2 years and a positive SPT to birch and/or timothy pollen. All patients were classified as having moderate to severe symptoms (itchy nose and eyes, sneezing, nasal secretion and nasal blockage) during birch and/or grass pollen season and they had all been treated with antihistamines and nasal steroids, often in combination with antihistamine or cromoglycate eye drops, during pollen seasons previous years. Patients had no symptoms of asthma at the time of visit and they did not take any asthma medication (short or long acting β-agonists and inhaled steroids). All patients presented 3+ or 4+ reactions towards birch or timothy in SPT. 13 patients presented positive reactions towards both birch and timothy and 5 patients were in addition positive for mugworth. Patients presenting positive reactions towards animals (4 towards cat, 2 towards dog and 2 towards horse), did not have any regular animal contact. Exclusion criteria included a history of perennial symptoms, upper airway infection during the last 2 weeks before the time of visit and treatment with local or systemic corticosteroids during the last 2 months. Occasional use of antihistamines was accepted.

The controls were all symptom-free, had no history of allergic rhinitis and had negative SPTs to the standard panel of allergens described above They had no history of upper airway infection during the last 2 weeks before the time of visit and they were all free of medication. Before inclusion, all subjects, patients as well as controls, were evaluated by an ear-, nose- and throat-consultant performing nasoscopy. Individuals with signs or symptoms of chronic rhinitis, hypertrophy of turbinates, septum deviation, nasal polyposis or recurrent sinusitis were excluded. None of the participants had been subjected to any form of nasal or sinus surgery before inclusion in the study. The study was approved by the Ethics Committee of the Medical Faculty, Lund University.

### Study design

Nasal biopsies for mRNA analysis were obtained from 12 patients with symptomatic seasonal allergic rhinitis, during either birch pollen (5 patients) or grass pollen season (7 patients). They were included when they had experienced substantial symptoms of rhinoconjunctivitis (itchy nose and eyes, sneezing, nasal secretion and nasal blockage) during at least 3 consecutive days. The majority of the patients were seen within 5–10 days after the first appearance of symptoms. A local pollen count confirmed the presence of the relevant pollen in the air during this period. In addition, 19 patients were seen before the pollen season and 18 healthy controls were sampled either before or during the pollen season.

Nasal biopsies for immunohistochemistry were obtained from 11 patients at two separate occasions outside pollen season. The first biopsy was obtained during control conditions without any form of challenge. 2–4 weeks later the same patients were challenged intranasally with relevant pollen. 24 hours after this challenge a second biopsy was obtained. Only one biopsy was taken from each nostril. Patients were challenged with 10,000 SQ/U per nostril of Aquagen (ALK, Denmark) with either birch (3 patients) or grass pollen (8 patients). 9 controls were sampled during the same period.

### Nasal biopsy procedure

Biopsies were taken from the inferior turbinate after topical application of local anesthesia containing lidocainhydrochloride/nafazoline (34 mg/mL/0.17 mg/mL) for 20 minutes. Biopsies were in total obtained from 69 different individuals. The nasal biopsies were approximately 2 × 2 × 2 mm large and too small to be divided without losing their morphology. As a consequence, material for mRNA characterization and immunohistochemistry could not be obtained from the same biopsy.

### RNA extraction and reverse transcription

Nasal biopsies for mRNA extraction were immediately placed in RNA-later (QIAGEN) for 24 hours and then frozen. RNA was extracted from homogenized nasal biopsies using the RNeasy Mini Kit (QIAGEN GmbH) according to the supplier's protocol. All samples were treated with DNase (QIAGEN). Total RNA quantity and quality were assessed by a spectrophotometer and the wavelength absorption ratio (260/280 nm) was between 1.8 and 2.0 in all preparations. Reverse transcription to cDNA was carried out with Omniscript™ reverse transcriptase kit (QIAGEN GmbH) with oligo-dT primer in a final volume of 20 μl using the Mastercycler personal PCR machine (Eppendorf AG, Germany), at 37°C for 1 hour.

### Quantitative real-time PCR

Quantitative real-time PCR assays were performed using the SmartCyclerII system (Cepheid, USA). Two different types of PCR assays were used, each with a different protocol according to the manufacturer's recommendations. For detection of TLR2 and β-actin, intron over-spanning oligonucleotide primers were designed (Table I) using Prime Express^® ^2.0 software (Applied Biosystems, USA) and synthesized by DNA Technology A/S (Aarhus, Denmark). PCR was performed using QuantiTect™SYBR^® ^Green PCR kit (QIAGEN) in a final volume of 25 μl. Reactions were incubated at 95°C for 15 min, then incubated 46 cycles at 94°C for 30 s followed by 55°C for 60 s (initially 65°C, followed by 2°C decrease the first 6 cycles). Standard curves for TLR2 and β-actin were prepared using half ^10^log dilutions of PCR products generated from target cDNA. Specific PCR products were analyzed by running melting curves. Melting curve analysis for TLR2 and β-actin revealed a single peak in each sample.

**Table 1:** Sequences of primers used for PCR amplification of TLR2 and β-actin.

Human TLR2	Forward, 5'-TCACTGCTTTCAACTGGTAGTTGTG-3'
	Reverse, 5'-TCCTTGGAGAGGCTGATGATG-3'
Human β-actin	Forward, 5'-GCCAACCGCGAGAAGATG-3'
	Reverse, 5'-ACGGCCAGAGGCGTACAG-3'

PCR: polymerase chain reaction

TLR: Toll-like receptor

For detection of TLR3, TLR4 and β-actin, primers were purchased from Applied Biosystems. These PCR assays contained a probe and were performed using Taq-Man^®^Universal PCR Master Mix NoAmpErase^®^UNG in a final volume of 25 μl. Reactions were incubated at 95°C for 10 min, then incubated 40 cycles at 95°C for 15 s followed by 60°C for 60 s. When using a probe as in these PCR assays, nonspecific amplification is not detected.

Gene expression changes were assessed using the comparative cycle threshold (C_T_) method . The relative amount of mRNA for TLR2, TLR3 and TLR4 was determined by subtracting the C_T _values achieved for these genes from the C_T _value of the housekeeping gene β-actin. The amount of mRNA is expressed in relation to 100,000 mRNA molecules of β-actin (100,000 × 2^-ΔC_T_^).

### Immunohistochemical analysis of TLRs

Nasal biopsies used for immunohistochemistry were frozen in Tissue Tek^® ^O.C.T mounting media (Histo Lab, Gothenburg, Sweden) immediately after excision. Cryosections, 8 μm thick, were after sectioning post-fixed with 2% buffered formaldehyde for 20 min, rinsed in phosphate buffered saline (PBS; pH 7.6; 3 × 5 min at room temperature (RT)) and placed in 0.1% saponin in PBS for 20 min at RT. Non-specific binding sites were blocked with 5% normal serum (Dako; dilution 1:10 in PBS) for 30 min. Avidin-binding sites were blocked with incubation of Avidin D solution (Vector Laboratories, Burlingame, CA, USA) for 15 min. Thereafter, the sections were rinsed in PBS (3 × 5 min) before blocking of biotin-binding sites with biotin blocking solution (Vector) for 15 min. After additional rinsing (PBS; 3 × 5 min) sections were incubated with the primary antibody overnight at 4°C (in control sections the primary antibody was omitted). The primary antibody was diluted in PBS supplemented with 0.25% Triton X and 0.25% bovine serum albumin. The primary antibodies were: anti-TLR2 (dilution 1:50), anti-TLR3 (dilution 1:100), anti-TLR4 (dilution 1:50). All primary antibodies were purchased from ImmunoKontact, AMS Biotechnology, Oxon, UK. After overnight incubation with primary antibody, the sections were rinsed (3 × 5 min in PBS) and incubated with biotinylated secondary antibody (horse anti-mouse IgG1, Vector, dilution 1:200 or goat anti-rabbit, dilution 1:200) for 45 min at RT. After additional rinsing (3 × 5 min in PBS), the sections were incubated with alkaline phosphatase-labeled Streptavidin (dilution 1:200 for 45 min), rinsed (3 × 5 min in PBS) and alkaline phosphate activity was developed for 6 min at RT using New Fuchsin (Dako) as enzyme substrate. Endogenous alkaline phosphatase activity was inhibited by Levamisol. All sections were counter-stained with Harris's hematoxylin, coated with Aqua Perm mounting medium (484975 Life Sci. International), dried overnight and mounted in DPX. Positive immunoreactivity was identified as bright red precipitate.

For quantification of TLR immunoreactivity, high resolution digital images were obtained from each biopsy, so that the entire mucosal area was captured. Occasional regions where the epithelial lining had been lost due to mechanical damage (such regions were present to an equal extent among the different groups) were excluded from the study. Using an image analysis system (Image-Pro Plus v4.51, Media Cybernetics, Silver Spring, USA) and a preset fixed colour threshold value, the number of positively (i.e. bright red) pixels was automatically calculated. The immunoreactivity of each TLR was expressed in relation to the area of mucosal tissue.

### Statistics

Statistical analysis was performed using GraphPad Prism 4. All data are expressed as mean ± SEM, and n equals the number of subjects. For mRNA data, group comparisons were made between controls and patients outside pollen season as well as between patients outside and during pollen season. For immunohistochemical data, group comparisons were made between controls and patients before allergen challenge as well as between patients before and after allergen challenge. Mann-Whitney test was used to determine statistical differences for unpaired comparisons and Wilcoxon signed rank test was used for paired comparisons. P-values less than or equal to 0.05 were considered statistically significant.

## Results

**Figure 1:**
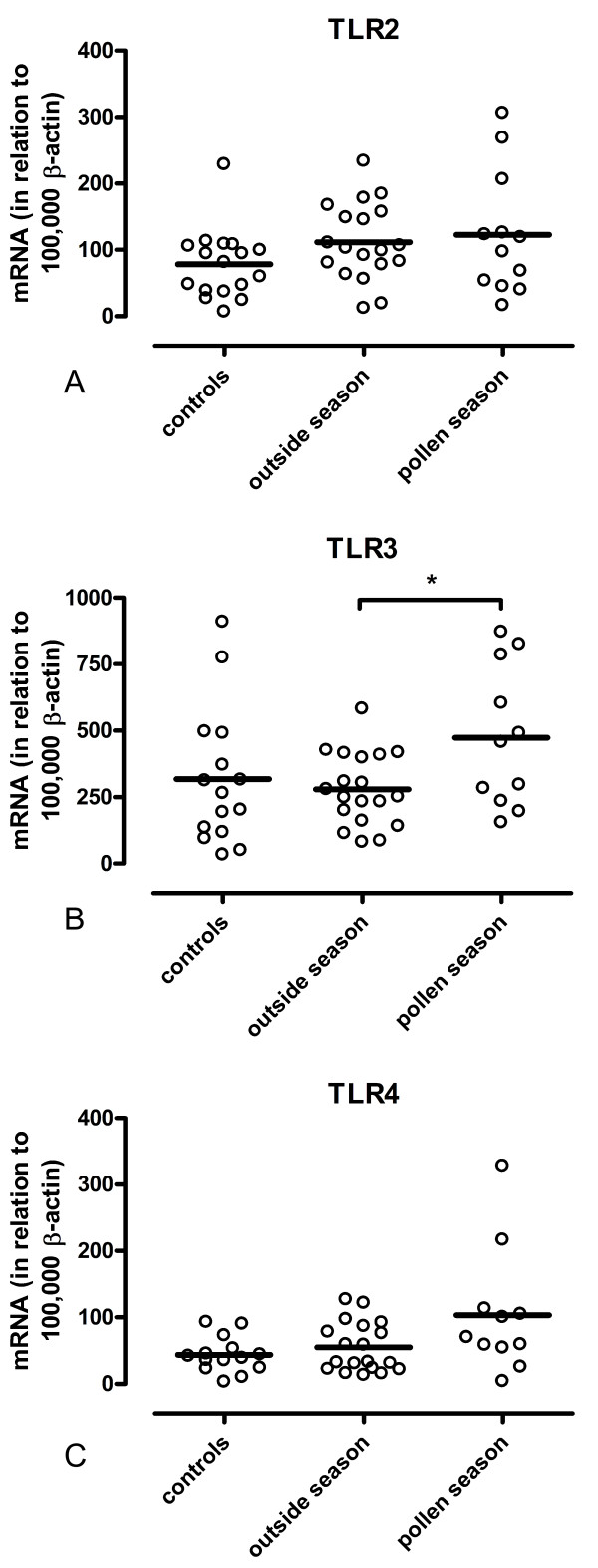
**mRNA expression of TLR2, TLR3 and TLR4 in nasal mucosa**. Expression levels of TLR2 (A), TLR3 (B) and TLR4 (C) in biopsies of nasal mucosa from healthy volunteers (controls), from patients with intermittent allergic rhinitis outside pollen season and from patients with symptomatic intermittent allergic rhinitis during pollen season. Levels of mRNA were calculated in relation to 100,000 mRNA molecules of β-actin. Bold lines represent mean values. Increase in the mRNA expression of TLR3 in patients during pollen season compared to patients outside pollen season (*p < 0.05).

Real-time PCR analysis of total RNA extracted from nasal biopsies demonstrated the presence of TLR2, TLR3 and TLR4 as well as β-actin in all samples. The expression of TLR2 mRNA, in relation to 100,000 molecules of β-actin, was 78 ± 13 in controls (n = 17), 112 ± 13 in patients outside pollen season (n = 19) and 122 ± 27 in patients during pollen season (n = 12). The differences seen between the groups were not statistically significant (Figure 1A). The expression of TLR3 mRNA was 318 ± 67 in controls (n = 15), 279 ± 31 in patients outside pollen season (n = 19) and 473 ± 80 in patients during pollen season (n = 11). The increase seen during pollen season was statistically significant (p < 0.05) (Figure 1B). The expression of TLR4 mRNA was 44 ± 7 in controls (n = 14), 55 ± 8 in patients outside pollen season (n = 19) and 103 ± 28 in patients during pollen season (n = 11). The apparent seasonal increase did not reach statistical significance (Figure 1C).

**Figure 2:**
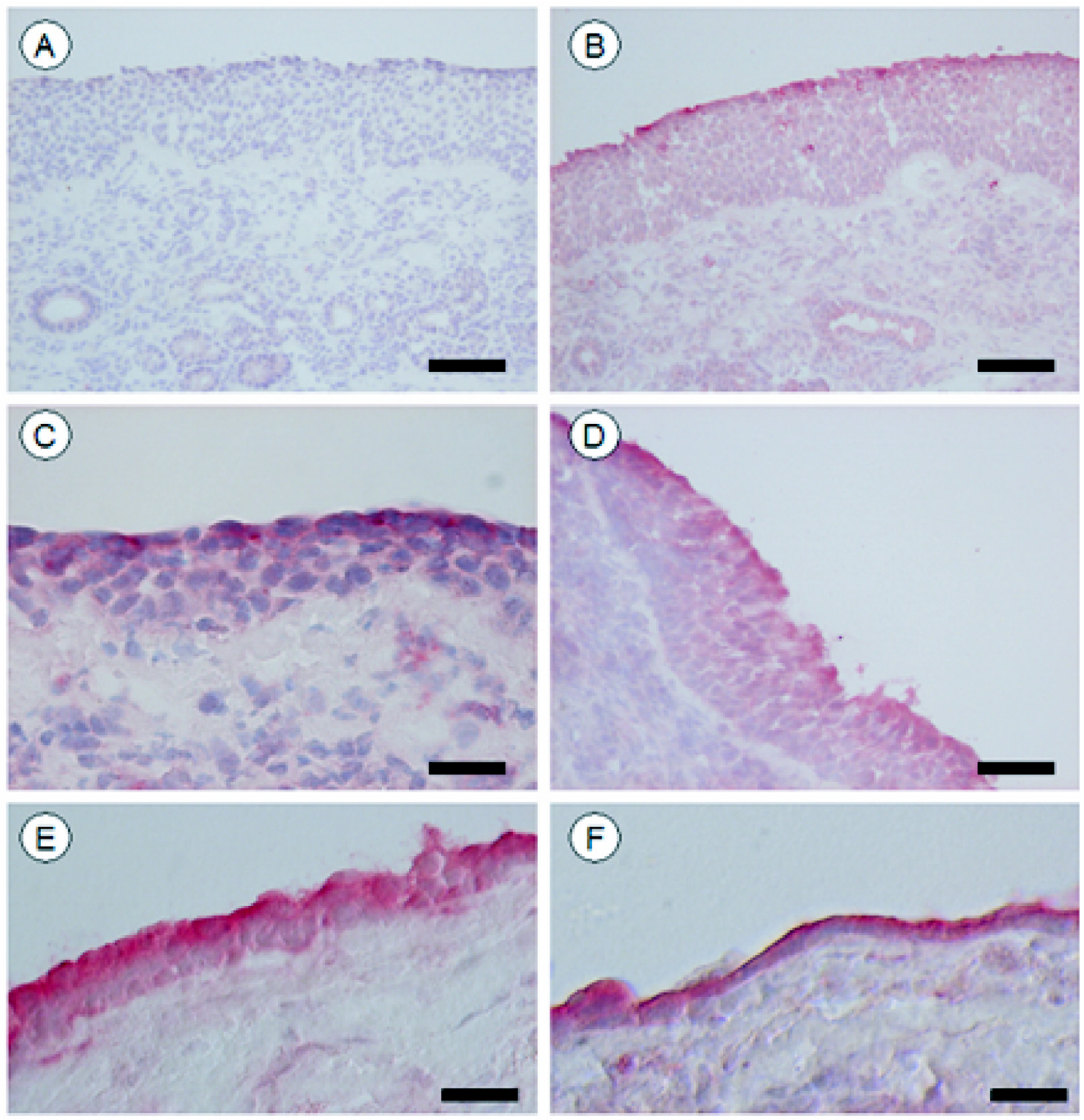
**Immunoreactivity of TLR2, TLR3 and TLR4 in nasal mucosa**. Immunohistochemical localization of TLR2, TLR3 and TLR4 in biopsies of nasal mucosa. Representative pictures of control (A) and TLR-stained (B-F) sections. No immunoreactivity was observed in control sections when the primary antibody was omitted (A). In an adjacent section, immunoreactivity for TLR3 is seen in the apical part of the epithelial lining and in a few scattered intraepithelial leukocytes (B). Pictures C and D illustrate representative staining patterns of TLR2 and TLR4, respectively. Areas with a thin repairing epithelium display a particularly intense staining, TLR3 (E) and TLR4 (F). Scale bars: A, B and D = 85 μm, C = 30 μm, E and F= 50 μm.

**Figure 3:**
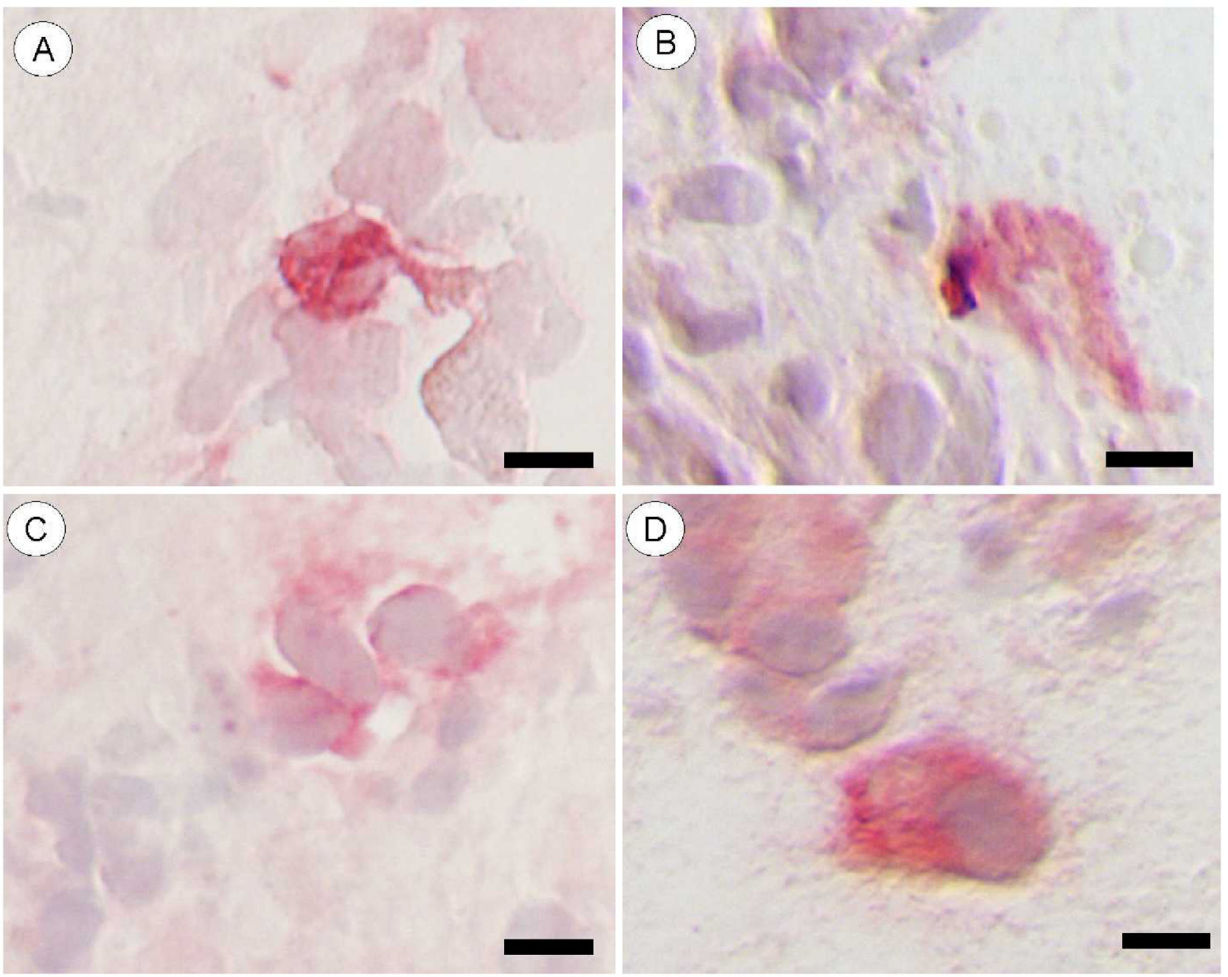
**Immunoreactivity of TLR2, TLR3 and TLR4 in subepithelial cells**. Bright field micrographs exemplifying TLR-positive cells in the subepithelial tissue. Granulocyte containing TLR2-like immunoreactivity (A), TLR3-positive mast cell (B), TLR4 immunoreactivity in lymphocyte-like cells (C) as well as in a large mononuclear cell (D). Scale bars: A, B and D = 10 μm, C = 8 μm.

All patients reported an increase in nasal symptoms both 5 and 15 min after allergen challenge (data not shown). The most intensive immunoreactivity for all three TLRs was seen within the airway epithelium (Figure 2A–F), where the staining was foremost distributed to epithelial cells positioned in the apical region of the epithelium. The same distribution pattern was observed among healthy controls and this pattern was not changed by the allergen challenge. A particularly intense staining was observed in scattered areas covered by a low-height repair epithelium. Immunoreactivity for the different TLRs was also seen in a few scattered intraepithelial leukocytes. For all examined TLRs, immunoreactivity was also present in scattered subepithelial cells. However, although being distinct from controls, the staining was generally variably pale and pallid which precluded a proper quantification. The weak intensity also jeopardized identification of positive cell types by e.g. double labeling techniques. Hence, a more tentative identification was made based on morphological criteria. In this regard, mast cells were identified as large granulated and mononuclear cells, granulocytes by their characteristic polymorph nuclei, lymphocytes as small mononuclear cells with a circular nucleus surrounded by only a thin rim of cytoplasm. Using these morphological criteria, TLR2 immunoreactivity could be observed in mast cells, granulocytes as well as in few scattered large non-granulated mononuclear cells (Figure 3A). Among the subepithelial TLR3-positive cells, only mast cells (Figure 3B) and occasional granulocytes were observed whereas immunoreactivity for TLR4 was present in mast cells, lymphocytes (Figure 3C), granulocytes and large non-granulated mononuclear cells (Figure 3D).

**Figure 4:**
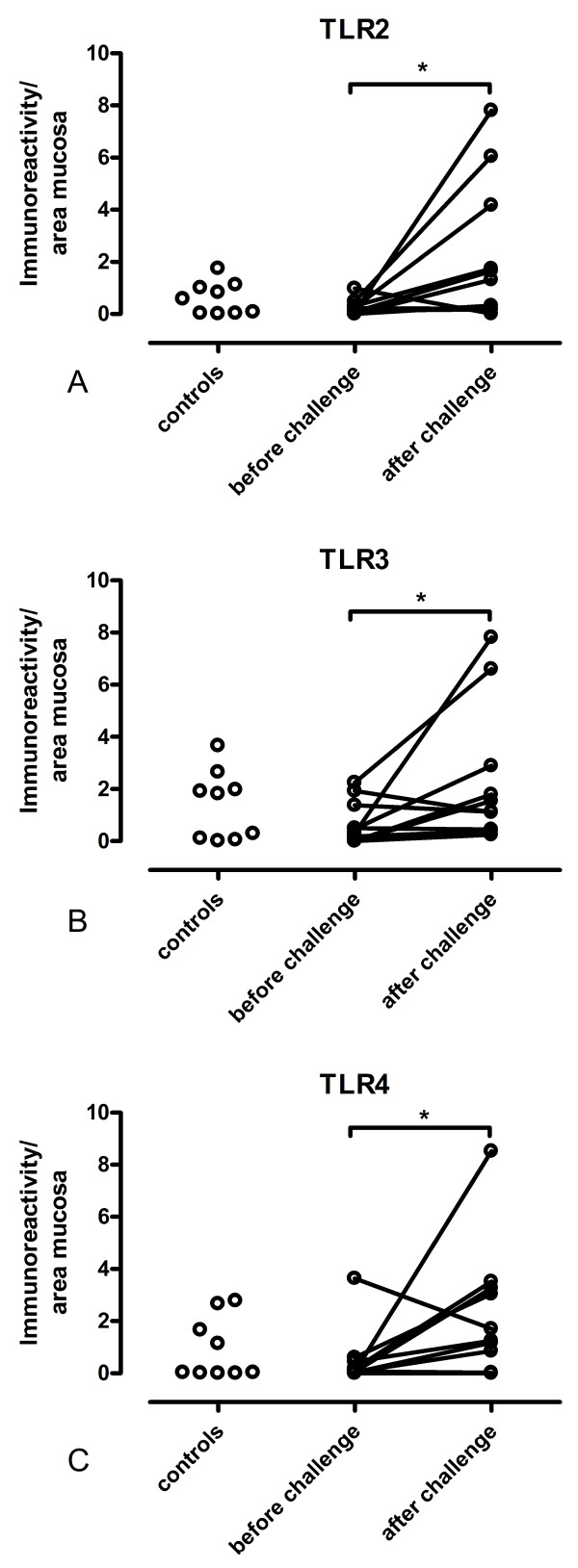
**Quantitative immunoreactivity of TLR2, TLR3 and TLR4 in nasal mucosa**. Quantitative analysis of TLR2 (A), TLR3 (B) and TLR4 (C) immunostaining in nasal epithelium in biopsies from healthy volunteers (controls), from patients with intermittent allergic rhinitis before nasal allergen challenge and from the same patients after allergen challenge. TLR immunoreactivity is expressed in relation to the area of mucosal tissue. Lines connecting patients before and after allergen challenge represent paired data. Increase in the immunostaining for TLR2 (*p < 0.05), TLR3 (*p = 0.05) and TLR4 (*p < 0.05) after allergen challenge.

The total immunoreactivity for TLR2, in relation to the area of mucosal tissue, was 0.61 ± 0.21 in controls (n = 9), 0.24 ± 0.29 in patients before allergen challenge (n = 11) and 2.16 ± 0.81 after allergen challenge (n = 11). There was an increase in TLR2 immunoreactivity after allergen challenge (p < 0.05; Figure 4A). Immunoreactivity for TLR3 was 1.39 ± 0.44 in controls (n = 9), 0.64 ± 0.25 in patients before allergen challenge (n = 11) and 2.22 ± 0.79 in patients after allergen challenge (n = 11). There was an increase in TLR3 immunoreactivity after allergen challenge (p = 0.05; Figure 4B). Immunoreactivity for TLR4 was 0.93 ± 0.40 in controls (n = 9), 0.47 ± 0.32 in patients before allergen challenge (n = 11) and 2.34 ± 0.80 in patients after allergen challenge (n = 10). There was an increase in TLR4 immunoreactivity after allergen challenge (p < 0.05; Figure 4C). 2 or 3 patients out of the 11 had a heightened response to allergen (Figure 4A–C). These patients were challenged with grass and with the exception of one patient, these data points all emanated from different individuals.

## Discussion

Real-time PCR demonstrated mRNA expression of TLR2, TLR3 and TLR4 in biopsies from the inferior turbinate of all tested subjects. Immunohistochemistry confirmed the occurrence of the corresponding proteins in the airway epithelium. Differences were not seen in mRNA content or in protein expression, when patients with seasonal allergic rhinitis were examined before pollen season or before allergen challenge and compared with healthy controls. A significant increase of mRNA for TLR3 was obtained during pollen season. The protein expression for all three TLRs increased in the rhinitis patients following a challenge with relevant pollen, supporting the idea of a role for TLRs in allergic airway inflammation.

The expression of TLRs has been established in different cells of the immune system, including dendritic cells, eosinophils, monocytes, macrophages and neutrophils, illustrating the role of TLRs in modulating inflammatory responses [19-21]. However, reports of protein verified TLR expression in human airways including nasal tissue are still lacking for most of the TLRs. In the present study, immunoreactivity for TLR2, TLR3 and TLR4 could be demonstrated in the apical part of the nasal epithelium. This is in line with previous findings for TLR2, TLR3 and TLR4 in cultured epithelial cells [4,14,22,23]. The sites for a possible TLR expression are known to vary between different cell types, i.e. TLR3 is found exclusively in the intracellular compartments of dendritic cells [24], whereas it can be found both on the cell surface and in intracellular parts of human lung fibroblasts [25]. Since TLRs are part of the first line of defense responding to the presence of different types of microbes in the airways, it seems reasonable that these receptors are localized in the apical part of the epithelial cells, facing the lumen. Expression of TLR4 has previously been described in the nasal mucosa from both children and adults irrespective of atopy status, and during control conditions this expression is more marked among children [17]. Only adults participated in the present study and it is unlikely that the slight difference in age between the patients [median age 36 (18–68)] and controls [median age 27 (16–50)] affected the outcome.

The amount or patterns of inflammatory cells with a positive staining for TLR2, TLR3 and TLR4 in the submucosa was limited and did not increase as a result of allergen challenge. Unfortunately the pallid appearance of the immunoreactivity in subepithelial cells precluded a proper identification of the TLR-positive cells (a potential explanation to the different intensity observed among epithelial and subepithelial cells may simply be that it reflects a relative higher expression i.e. more receptor molecules per cell within the epithelium). However, a tentative identification of subepithelial cells was made based on classical morphological criteria. Based on these it is surmised that TLR2 and TLR4 have a rather widespread expression among the immune cells, being present in mast cells, granulocytes, lymphocytes, and large agranular mononucleated cells (likely to be macrophages or dendritic cells). In contrast, subepithelial TLR3-like immunoreactivity was restricted to mast cells and occasional granulocytes.

The mRNA expression of TLR3 was found to be elevated among patients with on-going symptomatic allergic rhinitis. This is well in line with the increase of the corresponding protein seen following allergen challenge. mRNA for TLR4 exhibited a similar tendency, however not reaching statistical significance, whereas no seasonal increase was seen for TLR2 mRNA. The discrepancy between TLR2 and TLR4 mRNA on one hand and their corresponding proteins on the other could be explained in several ways. First of all, we must acknowledge that TLR2, TLR3 and TLR4 appear to be localized mainly in the apical part of the epithelium. mRNA from nasal biopsies is comprised of a heterogeneous mix of different cells not only from the epithelium, but also from the submucosa. An increase in TLR mRNA expression in the epithelial cells might therefore be masked by a lack of increase in the submucosal cells. Another explanation might be related to variations in the mRNA expression between individuals. The mRNA data were derived from two separate groups of patients, one sampled before and the other during pollen season. The protein data were paired, each patient contributed with two biopsies, one before and the other after allergen challenge, thereby omitting variations between individuals. Finally, a single allergen challenge differs from the prolonged allergen exposure seen during pollen season. It might be that the increase in immunoreactivity for TLR2, TLR3 and TLR4 seen after allergen challenge only persists for a limited period of time. If so, the mRNA expression might, after an initial increase, have decreased towards normal a few days into the pollen season.

The increased expression of TLR2, TLR3 and TLR4 as a consequence of allergen exposure is in line with a previous report demonstrating an increase of TLR2 protein in chronic middle ear inflammation [26]. The lack of increase in TLR2 and TLR4 mRNA, discussed above, is in accordance with another study demonstrating no differences in the expression of TLR2 and TLR4 when mRNA from normal turbinates were compared with samples from patients with chronic sinusitis and/or nasal polyps [3]. Proteins were not investigated in the latter study. It is also worth noticing that the inflammation in chronic sinusitis and nasal polyps is different from the acute inflammation seen in the nasal epithelium during intermittent allergic rhinitis.

The role of TLRs as a primary part of our microbe defense system has been shown in several studies, but their possible function as mediators in allergy and asthma remains to be established. Thus, their role as detectors of pathogens has provided molecular mechanisms to underpin the observations leading to the so-called hygiene hypothesis. This theory proposes that a major source of microbial, Th1-like, immune provocations has been lost with the decreased incidence of many infectious diseases due to vaccinations, the use of antibiotics, and a higher hygiene standard [27]. The deficiency in Th1-like provocations leads to Th2-biased immune responses towards environmental allergens and consequently to an increase in allergic airway diseases [28,29]. In line with this hypothesis, it has been shown that children of farmers, known to have a decreased risk of developing allergies, expressed higher levels of TLR2 mRNA in blood compared to children of non-farmers. The expression of TLR4 was not altered in farmers' children, but the level of the co-factor CD14 was markedly increased, indicating a change in receptor mediated signaling activity [29]. However, once the allergic airway inflammation has been established, bacterial and viral infections may be as relevant as allergens in inducing hyperreactive responses [30-33].

## Conclusion

An up-regulation of TLR2, TLR3 and TLR4 in the nasal mucosa of patients with symptomatic allergic rhinitis supports the idea of a role for TLRs in allergic inflammation. In several airway model systems, stimulation of TLRs results in changes in the production of effector molecules, such as cytokines and chemokines, thereby affecting and further upgrading the airway inflammation [4,14-17]. It is also well known that viral as well as bacterial infections can worsen the symptoms of allergic rhinitis and trigger exacerbations of asthma [6]. Thus, an increase in the amount of TLRs present in the apical part of the epithelium might induce a hyperreactive response to bacteria and viruses in patients with seasonal allergic rhinitis.

## List of abbreviations used

PCR: polymerase chain reaction

RT: room temperature

SPT: skin prick test

TLR: Toll-like receptor

## Competing interests

The study was financially supported by the Swedish Medical Research Council, the Swedish Heart Lung Foundation, the Swedish Association for Allergology and AstraZeneca, Sweden.

Lennart Jansson is employed by AstraZeneca R&D.

## Authors' contributions

MF acquired and analyzed the mRNA data and drafted the manuscript. MA acquired and analyzed the mRNA data and revised the content of the manuscript. JE and RU acquired and analyzed the immunohistochemistry data and revised the content of the manuscript. LJ contributed to the study-design and revised the data of the manuscript. LOC conceived of the study, participated in its design and coordination, and helped to draft the manuscript

## References

[B1] Janeway CA, Medzhitov R (2002). Innate immune recognition. Annu Rev Immunol.

[B2] Vandermeer J, Sha Q, Lane AP, Schleimer RP (2004). Innate immunity of the sinonasal cavity: expression of messenger RNA for complement cascade components and toll-like receptors. Arch Otolaryngol Head Neck Surg.

[B3] Claeys S, de Belder T, Holtappels G, Gevaert P, Verhasselt B, van Cauwenberge P, Bachert C (2003). Human beta-defensins and toll-like receptors in the upper airway. Allergy.

[B4] Sha Q, Truong-Tran AQ, Plitt JR, Beck LA, Schleimer RP (2004). Activation of airway epithelial cells by toll-like receptor agonists. Am J Respir Cell Mol Biol.

[B5] Sabroe I, Read RC, Whyte MK, Dockrell DH, Vogel SN, Dower SK (2003). Toll-like receptors in health and disease: complex questions remain. J Immunol.

[B6] Zuany-Amorim C, Hastewell J, Walker C (2002). Toll-like receptors as potential therapeutic targets for multiple diseases. Nat Rev Drug Discov.

[B7] O'Neill LA (2003). Therapeutic targeting of Toll-like receptors for inflammatory and infectious diseases. Curr Opin Pharmacol.

[B8] Heine H, Lien E (2003). Toll-like receptors and their function in innate and adaptive immunity. Int Arch Allergy Immunol.

[B9] Sabroe I, Parker LC, Wilson AG, Whyte MK, Dower SK (2002). Toll-like receptors: their role in allergy and non-allergic inflammatory disease. Clin Exp Allergy.

[B10] Cario E, Podolsky DK (2000). Differential alteration in intestinal epithelial cell expression of toll-like receptor 3 (TLR3) and TLR4 in inflammatory bowel disease. Infect Immun.

[B11] Pitzurra L, Bellocchio S, Nocentini A, Bonifazi P, Scardazza R, Gallucci L, Stracci F, Simoncelli C, Bistoni F, Romani L (2004). Antifungal immune reactivity in nasal polyposis. Infect Immun.

[B12] Muir A, Soong G, Sokol S, Reddy B, Gomez M, Van Heeckeren A, Prince A (2003). Toll like receptors in normal and cystic fibrosis airway epithelial cells. Am J Respir Cell Mol Biol.

[B13] Guillot L, Medjane S, Le-Barillec K, Balloy V, Danel C, Chignard M, Si-Tahar M (2004). Response of human pulmonary epithelial cells to lipopolysaccharide involves Toll-like receptor 4 (TLR4)-dependent signaling pathways: evidence for an intracellular compartmentalization of TLR4. J Biol Chem.

[B14] Monick MM, Yarovinsky TO, Powers LS, Butler NS, Carter AB, Gudmundsson G, Hunninghake GW (2003). Respiratory syncytial virus up-regulates TLR4 and sensitizes airway epithelial cells to endotoxin. J Biol Chem.

[B15] Hertz CJ, Wu Q, Porter EM, Zhang YJ, Weismuller KH, Godowski PJ, Ganz T, Randell SH, Modlin RL (2003). Activation of toll-like receptor 2 on human tracheobronchial epithelial cells induces the antimicrobial peptide human beta defensin-2. J Immunol.

[B16] Armstrong L, Medford AR, Uppington KM, Robertson J, Witherden IR, Tetley TD, Millar AB (2004). Expression of functional toll-like receptor-2 and -4 on alveolar epithelial cells. Am J Respir Cell Mol Biol.

[B17] Tulic MK, Fiset PO, Manoukian JJ, Frenkiel S, Lavigne F, Eidelman DH, Hamid Q (2004). Role of toll-like receptor 4 in protection by bacterial lipopolysaccharide in the nasal mucosa of atopic children but not adults. Lancet.

[B18] Aas K, Belin L (1973). Standardization of diagnostic work in allergy. Int Arch Allergy Appl Immunol.

[B19] Muzio M, Bosisio D, Polentarutti N, D'Amico G, Stoppacciaro A, Mancinelli R, van't Veer C, Penton-Rol G, Ruco LP, Allavena P (2000). Differential expression and regulation of toll-like receptors (TLR) in human leukocytes: selective expression of TLR3 in dendritic cells. J Immunol.

[B20] Kaisho T, Akira S (2003). Regulation of dendritic cell function through Toll-like receptors. Curr Mol Med.

[B21] Underhill DM (2003). Toll-like receptors: networking for success. Eur J Immunol.

[B22] Soong G, Reddy B, Sokol S, Adamo R, Prince A (2004). TLR2 is mobilized into an apical lipid raft receptor complex to signal infection in airway epithelial cells. J Clin Invest.

[B23] Greene CM, Carroll TP, Smith SG, Taggart CC, Devaney J, Griffin S, O'Neill SJ, McElvaney NG (2005). TLR-induced inflammation in cystic fibrosis and non-cystic fibrosis airway epithelial cells. J Immunol.

[B24] Matsumoto M, Funami K, Tanabe M, Oshiumi H, Shingai M, Seto Y, Yamamoto A, Seya T (2003). Subcellular localization of Toll-like receptor 3 in human dendritic cells. J Immunol.

[B25] Matsumoto M, Kikkawa S, Kohase M, Miyake K, Seya T (2002). Establishment of a monoclonal antibody against human Toll-like receptor 3 that blocks double-stranded RNA-mediated signaling. Biochem Biophys Res Commun.

[B26] Shuto T, Imasato A, Jono H, Sakai A, Xu H, Watanabe T, Rixter DD, Kai H, Andalibi A, Linthicum F (2002). Glucocorticoids synergistically enhance nontypeable Haemophilus influenzae-induced Toll-like receptor 2 expression via a negative cross-talk with p38 MAP kinase. J Biol Chem.

[B27] Strachan DP (1989). Hay fever, hygiene, and household size. Bmj.

[B28] Warner JO (1999). Worldwide variations in the prevalence of atopic symptoms: what does it all mean?. Thorax.

[B29] Lauener RP, Birchler T, Adamski J, Braun-Fahrlander C, Bufe A, Herz U, von Mutius E, Nowak D, Riedler J, Waser M (2002). Expression of CD14 and Toll-like receptor 2 in farmers' and non-farmers' children. Lancet.

[B30] (2002). Use of antibiotics to treat asthma exacerbations. J Allergy Clin Immunol.

[B31] Skoner DP (2002). Viral infection and allergy: lower airway. Allergy Asthma Proc.

[B32] Michel O (2001). Role of house-dust endotoxin exposure in aetiology of allergy and asthma. Mediators Inflamm.

[B33] Blease K, Kunkel SL, Hogaboam CM (2001). IL-18 modulates chronic fungal asthma in a murine model; putative involvement of Toll-like receptor-2. Inflamm Res.

